# Thermal Behavior of Water in Sephadex^®^ G25 Gels at Low Temperatures Studied by Adiabatic Calorimetry

**DOI:** 10.3390/gels9020126

**Published:** 2023-02-02

**Authors:** Noriko Onoda-Yamamuro, Hiroaki Minato, Eiji Nakayama, Norio Murase

**Affiliations:** 1 Division of Science, School of Science and Engineering, Tokyo Denki University, Hatoyama, Hiki-gun, Saitama 350-0394, Japan; 2 School of Science and Engineering, Tokyo Denki University, Hatoyama, Hiki-gun, Saitama 350-0394, Japan

**Keywords:** Sephadex^®^ G25, gel, ice crystallization, glass transition, enthalpy relaxation, adiabatic calorimetry

## Abstract

Water in a crosslinked dextran gel, Sephadex^®^ G25, is known to remain partially unfrozen during cooling and undergoes ice crystallization during rewarming. However, the mechanism of ice crystallization during rewarming is still unclear. To elucidate the frozen state of water in the gel, thermal behavior at low temperatures was investigated by using adiabatic calorimetry. Heat capacities and enthalpy-relaxation rates of the gel-containing water of mass ratio *h* (=g H_2_O/g dry G25) = 1.00 were measured between 80 and 300 K during rewarming, where the gel was intermittently heated at the rate of 50–100 mK min^−1^. Although an exotherm indicating ice crystallization during rewarming was confirmed with the gel precooled rapidly, at 5 K min^−1^, it disappeared when precooled slowly, at 20 mK min^−1^. During rewarming after the rapid cooling, two glass transitions were observed at ca. 175 K and 240–242 K. A higher-temperature glass transition due to the water trapped by the polymer network was not so clear, as it was overlapped with an endotherm due to the melting of small ice crystals, which indicates that the ice crystals formed have a broad size-distribution and that water in the gel is vitrified when ice crystals of even the smallest size cannot be formed.

## 1. Introduction

Ice crystallization in polymer gels reflects the characteristics of the gels, such as the flexibility of the polymer chains and the network structure of the gels. Freezing behavior of crosslinked polymer gels has been studied, and interest in the ice crystallization in gels itself has grown. From these studies, water in polymer gels was defined as compartmentalized water, which means the state of water between continuous and discrete existing in the polymer-network structure [1,2,3,4,5,6,7].

Water in crosslinked dextran gels of a certain crosslink density, Sephadex^®^ G25, is known to remain partially unfrozen during precooling and undergoes ice crystallization during rewarming (heating after cooling). Ice crystallization during rewarming has been investigated mainly by DSC (differential scanning calorimetry) [1,2,3,4,5,6,7]. It was found that it is due to the water trapped by the polymer network at the time of freeze initiation during precooling [4]. Using various physical techniques, including XRD (X-ray diffraction) [6,7], X ray-CT (computed tomography) [7], and Raman spectroscopy [5], the frozen state of water in the gel has been intensively studied by one of the present authors [1,2,3,4,5,6,7]. As regards the conditions for the observation of ice crystallization during rewarming within the gel, the appearance of an exotherm in a DSC rewarming trace, they were summarized into several factors, i.e., density of crosslinks, water content, cooling rate, initial temperature of ice crystallization during precooling, etc. However, the mechanism of the ice crystallization is still unclear.

In addition to the exotherm in the DSC rewarming trace for the Sephadex^®^ G25 gels, an endothermic trend appears just before the exotherm. If we assume that the extent of the trend corresponds to the change in heat capacity due to a glass transition, the value calculated, ~100 J K^−1^g^−1^ [3], is too large compared with that obtained for the vapor-condensed glassy water, 1.94 J K^−1^g^−1^ [8], and glucose, a constituent of dextran, 0.65 J K^−1^g^−1^ [9].

There is a possibility that the origin of the endothermic trend is due to the melting of small ice crystals, the melting temperature of which is depressed by the Gibbs–Thomson effect [3,7]. In fact, the presence of small ice crystals less than ~10 µm in diameter was indicated from the 2D-XRD pattern with the Sephadex^®^ G25 gel [6]. Ice grains, detected with the gel using X-ray CT, were 3–5 µm in diameter and became larger than 10 µm in diameter after the occurrence of ice crystallization during rewarming [7]. However, small ice crystals of ~nm size, estimated by using the interfacial energy between ice and bulk water [3], have not been detected.

Clarification of the origin of the endothermic trend is a prerequisite for understanding the mechanism of ice crystallization during rewarming. Moreover, it is also necessary to make clear the presence and the temperature of glass transitions for the understanding.

In this connection, the temperature of glass transition (*T*_g_) for the confined water in silica MCM-41 nanopores was obtained by using adiabatic calorimetry [10]. *T*_g_ became higher with the growth of the assembly of water molecules in the nanopores by strengthening the hydrogen-bond network, and 210 K was suggested as *T*_g_ for the bulk water. It is higher than the *T*_g_ obtained with the hyper-quenched liquid water, 138 ± 1 K [11]. *T*_g_ for the water in the Sephadex^®^ G25 gel, if existing below the temperature of ice crystallization during rewarming, around 260 K, might be of interest in the context of water science [12] as well as the characterization of crosslinked dextran-forming gels [4,13].

Adiabatic calorimetry is a very powerful tool for the analysis of the frozen state, including the detection of glass transitions accompanying relaxation phenomena [10,14,15]. In this study, therefore, the thermal behavior of Sephadex^®^ G25 gels at low temperatures was investigated by using adiabatic calorimetry together with DTA (differential thermal analysis).

## 2. Results and Discussion

### 2.1. DTA Measurement

**Figure 1:**
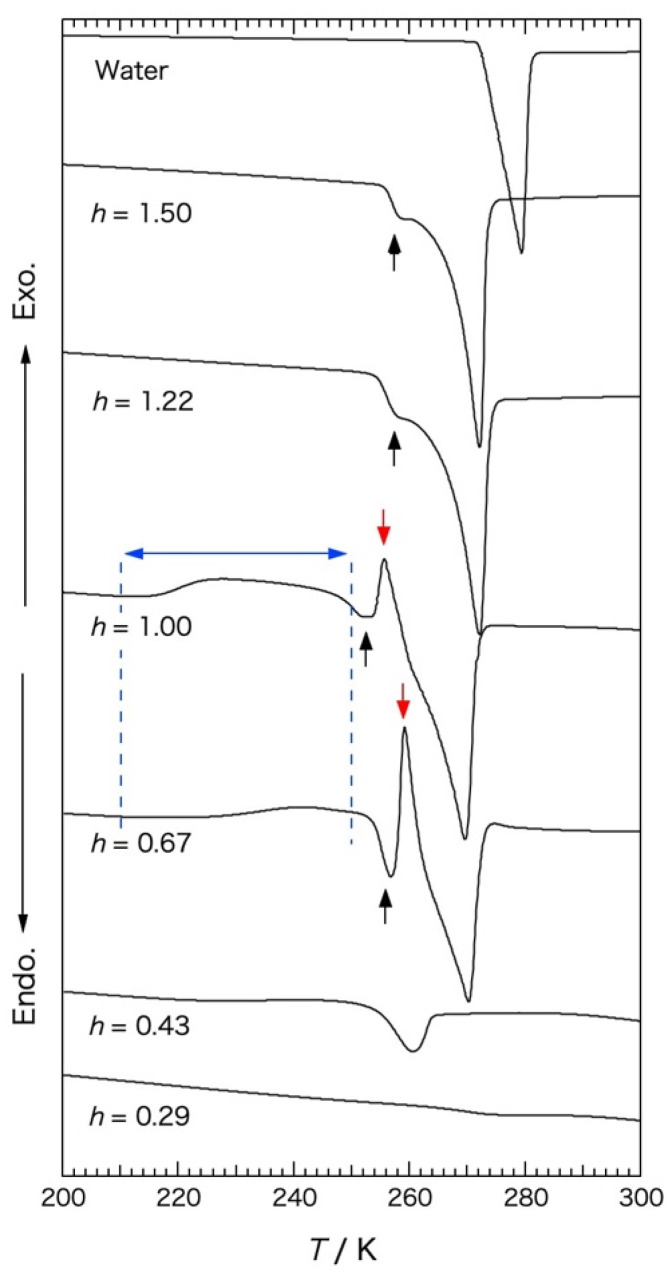
DTA rewarming traces obtained with Sephadex^®^ G25 gels (*h* = 0.29–1.50), where *h* = g H_2_O/g dry G25. Heating rate was 2 K min^−1^ and precooling rate was 5 K min^−1^. The vertical scale was set arbitrarily in each thermogram. ↑: small endotherm, ↓: exotherm due to ice crystallization, ↔: temperature range of exotherm.

DTA rewarming traces obtained with G25 gels containing water of mass ratio *h* = 0.29–1.50 are shown in Figure 1, where *h* = g H_2_O/g dry G25. The heating rate was 2 K min^−1^ and the rate of the previous cooling was 5 K min^−1^. The vertical scale was set arbitrarily in each thermogram to make it easy to compare the thermograms.

With pure water used as the calibration of the handmade apparatus, an endotherm due to the melting of ice was observed at 273 K. A small endotherm indicated by black arrows was observed with the gels containing water of mass ratio *h* ≥ 0.67 at a lower temperature than that of the large endotherm due to the melting of ice. The initiation temperature of the small endotherm was ca. 252 K for the gels of *h* = 0.67 and 1.00 and ca. 254 K for the gels of *h* = 1.22 and 1.50. The origin of the small endotherm was the melting of small ice crystals, the melting temperature of which was depressed by the Gibbs–Thomson effect [3,7]. This was demonstrated by XRD and X ray-CT studies [6,7]. The gel samples of *h* = 0.67 and 1.00 indicated an exotherm due to ice crystallization at a temperature several degrees above that of the melting endotherm of the small ice crystals, indicated by red arrows. The gel samples of *h* = 1.22 and 1.50 did not show such an exotherm, which became smaller with the increase in water content. This behavior can be explained by considering the swelling of the network structure of the polymer gel with the increase in the water content and appearance of the bulk-like water inside and outside the gel beads [16]. In the case of Sephadex^®^ G15 gels, it was reported that the exotherm due to the ice crystallization during rewarming was not observed in the DSC traces regardless of the *h* value [2,17]. As the network structure of the Sephadex^®^ G15 is hard to change at the time of initiation of ice crystallization because of the higher crosslink density than that of the G25 gel, water in the G15 gel is difficult to trap by the network structure and turn into a glassy state [2]. With the G25 gel sample of *h* = 0.43, the initiation temperature of the ice melting was ca. 254 K, which was due to the trapped water existing discretely in the network structure. However, no exotherm was observed during rewarming, which is because the network structure hardly changed at the time of the initiation of ice crystallization during precooling and the trapped water did not turn into a glassy state as the network structure was not swollen and was not as flexible. With the G25 gel sample of *h* = 0.29, no endotherm due to the melting of ice was observed. These results coincide well with the results previously reported [1]. Different from these results obtained by previous studies, an exotherm of the broad temperature range of 210–250 K was observed for the first time in this study with the gel samples of *h* = 0.67–1.00.

### 2.2. Adiabatic Calorimetry

**Figure 2:**
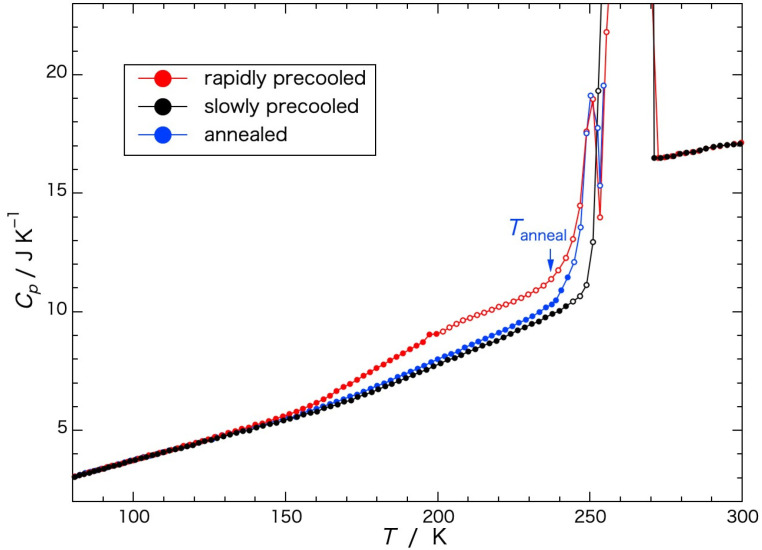
Heat capacities of a Sephadex^®^ G25 gel (*h* = 1.00) dependent on the rate of previous cooling. ●: rapidly precooled, ●: slowly precooled, ●: annealed. Open circles represent the data obtained before reaching equilibrium. *T*_anneal_ indicates a temperature of annealing.

Heat capacities of a Sephadex^®^ G25 gel (*h* = 1.00) dependent on the rate of previous cooling are shown in Figure 2, wherein the apparent heat capacities obtained when crystallization or melting was in progress and the equilibrium state was not reached are indicated by open circles. The heat capacities (*C_p_*) of the rapidly precooled Sephadex^®^ G25 gel (red circle) began to increase gradually in temperature from 150 K to 200 K, increasing steeply from around 240 K, and ice melting began at 245 K. Above the temperature of the initiation of ice melting, an exotherm due to ice crystallization appeared at 250–255 K. The endotherm due to ice melting finished at 271 K. In the case of the gel annealed at 237 K, the temperature below the initiation of the steep increase in the heat capacity, for 13 h (blue circle) followed by rapid cooling the heat capacity increased in a smooth curve from 80 K to the annealing temperature. Immediately above the annealing temperature, a steep increase occurred followed by the exotherm due to ice crystallization at 250–255 K, similar to the rapidly precooled gel. In the case of the gel precooled slowly (black circle), the heat capacity increased in a smooth curve until the temperature of ice melting, showing no exotherm in the temperature range from 80 K to 273 K.

**Figure 3:**
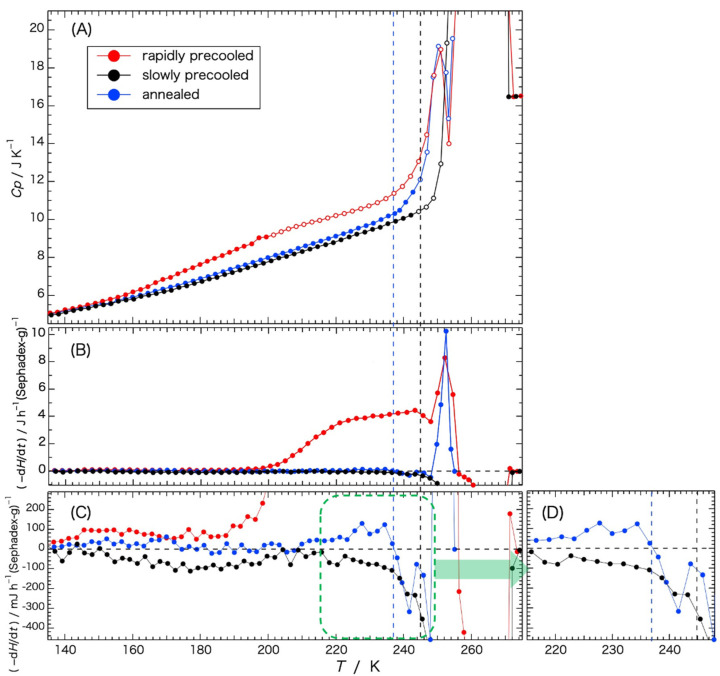
Rates of the enthalpy change –(d*H*/d*t*) dependent on the rate of previous cooling –(d*H*/d*t*) > 0: enthalpy release; –(d*H*/d*t*) < 0: enthalpy absorption. ●: rapidly precooled, ●: slowly precooled, ●: annealed. Data of the heat capacity shown in Figure 2 are redisplayed in (**A**) for reference. A vertical enlargement of (**B**) is shown in (**C**). A lateral enlargement of the green dashed-line area in (**C**) is shown in (**D**). The vertical dashed line in blue represents the annealing temperature and the vertical dashed line in black the initiation temperature of ice melting.

Temperature dependence of the rates of enthalpy change –(d*H*/d*t*) observed during the equilibration period in the heat-capacity measurement is shown in Figure 3. The quantities were expressed by –(d*H*/d*t*) = *C*_gross_(d*T*/d*t*), where *C*_gross_ is the gross heat capacity of the cell containing the sample. The rate (d*T*/d*t*) of the temperature increase was measured 5 min after each energy input. The time (5 min) was sufficiently longer than the thermal-equilibration time. In Figure 3, data of the heat capacity shown in Figure 2 are redisplayed for reference (Figure 3A). Figure 3C is a vertical enlargement of Figure 3B. Figure 3D is a lateral enlargement of the green dashed-line area in Figure 3C.

**Figure 4:**
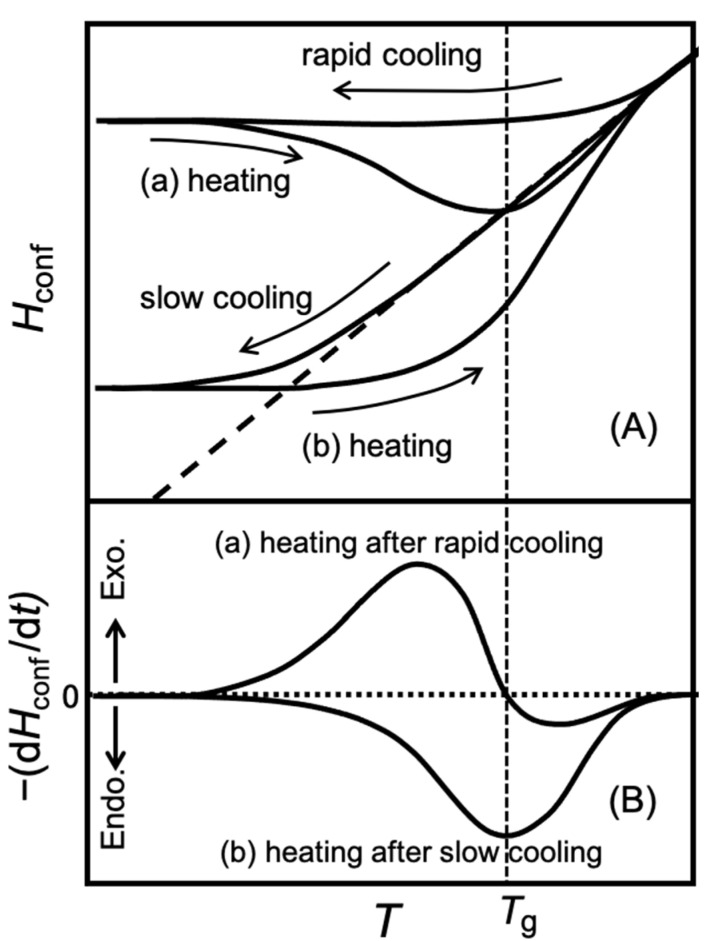
Schematic presentation of typical temperature dependence of the configurational enthalpy *H*_conf_ of the molecule near the glass-transition temperature (**A**) and the spontaneous enthalpy-relaxation rate –(d*H*_conf_/d*t*) observed in the heat-capacity measurement (**B**) [10]. The dashed line in (**A**) indicates the equilibrium enthalpy. The vertical dashed line indicates the glass-transition temperature *T*_g_.

Rates of the enthalpy relaxation dependent on the rate of the previous cooling are shown in Figure 3B,C. In the temperature range from 130 K to 190 K, the spontaneous enthalpy-relaxation rate (Figure 3C) is shown with the pair of the gels precooled rapidly and indicated a behavior characteristic of slow glass transition, as explained in Figure 4B. From the temperature of 200 K to 244 K, the rates of enthalpy change observed with the rapidly precooled gel (Figure 3B) indicated a remarkable enthalpy-release effect, which corresponds to the exotherm observed in the temperature range from 210 K to 250 K in Figure 1. This is due to the ice crystallization following the glass transition. It is true that the pattern of the spontaneous enthalpy-relaxation rate shown in Figure 4B was for the sample showing no crystallization in the neighborhood of the glass-transition temperature. However, most of the water in the G25 gel precooled slowly had crystallized during cooling and hardly any enthalpy-release effect was observed (Figure 3B), similar to the annealed gel precooled from the annealing temperature (237 K) that was already enthalpy-released during the annealing (see Figure 4 for details). Taking these into consideration, i.e., spontaneous enthalpy release (130–190 K) and remarkable enthalpy release (200–244 K) observed with the rapidly precooled G25 gel, it can be concluded that the gradual increase in the heat capacity in the range from 150 K to 200 K in the rapidly precooled sample, shown in Figure 3A, was a glass transition from a vitreous state to a supercooled liquid state, and that the exothermic effect observed in the broad temperature range from 200 K to 244 K in Figure 3B was due to the ice crystallization proceeding gradually. The glass-transition temperature was estimated to be ca. 175 K.

The decrease in the exothermic effect at 245–248 K in the rapidly precooled sample (Figure 3B) was caused by the overlapping of the enthalpy-absorption effect due to the melting of small ice crystals. Then, the rate of the enthalpy change showed an abrupt enthalpy release due to ice crystallization at 250–255 K, followed by the melting of the ice finishing at 271 K.

In the case of the annealed gel, the enthalpy-release effect observed with the rapidly precooled gel in the range from 200 K to 244 K in Figure 3B disappeared. This means that the supercooled liquid water crystallized during the annealing treatment. The second remarkable enthalpy-release effect due to the ice crystallization at 250–255 K, observed with the rapidly precooled sample, remained also with the annealed gel. Looking at the enlarged figure (Figure 3D), rates of the enthalpy relaxation indicated small positive values of the enthalpy-release effect from 210 K to the annealing temperature at 237 K, where the relaxation turned to negative values. Considering the enthalpy-relaxation behavior changing from a positive to a negative value and the succeeding exotherm due to ice crystallization, the steep increase in the heat capacity starting at 240–242 K (Figure 3A) indicates a second (higher) glass transition. The heat capacity increased further because of the initiation of ice melting at 245 K (black dashed line in Figure 3). The temperature of the second glass transition was estimated to be 240–242 K.

**Figure 5:**
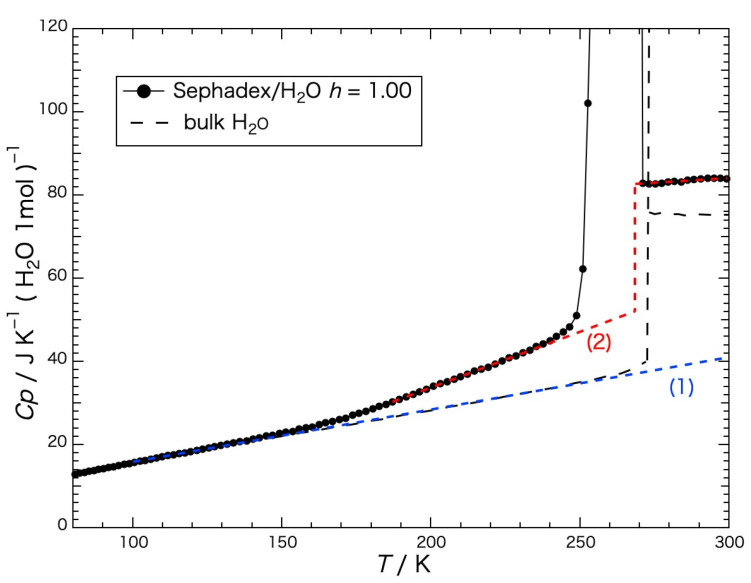
Heat capacities of the water per mole within the Sephadex^®^ G25 gel (*h* = 1.00) precooled slowly. A black dashed line indicates the heat capacity of the bulk H_2_O (water or ice) [18]. The blue dashed line (1) represents the baseline for determining the configurational enthalpy, and the red dashed line (2) represents the baseline for determining the fusion enthalpy (see text for details).

The heat capacity of the water per mole within the gel precooled slowly is shown in Figure 5, and was obtained by subtracting the heat-capacity values of the dried gel from those of the gel containing water. The black dashed line in the figure indicates the heat capacity of the bulk H_2_O (water or ice) [18]. The melting temperature was 273.15 K. The blue dashed line (1) represents the baseline for determining the configurational enthalpy and the red dashed line (2) represents the baseline for determining the fusion enthalpy. The configurational enthalpy *H*_conf_ is the enthalpy due to the spatial rearrangement of molecules. The heat capacity of the bulk ice, which mainly represents that of the vibrational motion without contribution of the rearrangement of the water molecules, increased with the temperature, and the motion of the configurational change occurred instantaneously at the melting temperature. Although the heat capacity of the water in the gel increased similarly to that of the bulk ice until reaching a temperature of ca. 150 K, it began to increase more than that of the bulk ice above the temperature, where the change in configuration gradually started and jumped up at around the melting temperatures as a first-order phase transition.

**Figure 6:**
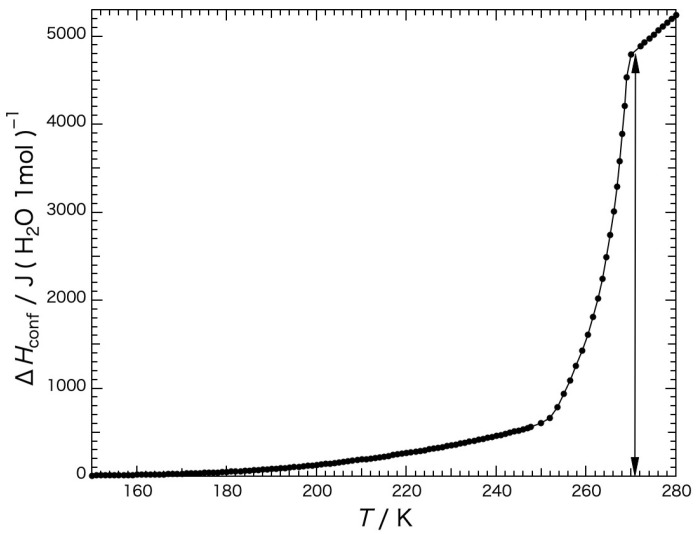
Temperature dependence of the configurational enthalpy of the water within the Sephadex^®^ G25 gel (*h* = 1.00) precooled slowly.

The temperature dependence of the configurational enthalpy of the water within the Sephadex^®^ G25 gel precooled slowly is shown in Figure 6, where the enthalpy was obtained up to 248 K by the integration of the heat-capacity difference between that of the water within the gel and that of the bulk ice. The heat capacity of the bulk ice was approximated by a straight line connecting the *Cp* values at 150 K and 230 K and extrapolated to higher temperatures (see the blue dashed line (1) in Figure 5). In the first-order phase-transition-temperature region from 248 K to 270 K, the enthalpy was obtained by summing up the input energy corrected for the effect of slight heat leakage.

As shown in Figure 6, it was found that the configurational enthalpy in the gel began to increase from 150 K, and that from 150 K to the temperature of the completion of ice melting (271 K) the increase in the enthalpy was 4.80 kJ (mol H_2_O)^−1^, which corresponds to ca. 80% of the configurational enthalpy, i.e., the fusion enthalpy of ice, 6.01 kJ mol^−1^). One of the reasons for the configurational enthalpy of the water within the gel being small is because the change in configuration of the water within the gel was not as free just above the melting temperatures compared with that of the bulk water. As the heat capacity of the water within the gel was 11% larger above the melting temperatures than that of the bulk water, the rate of the configurational change of the water within the gel rapidly approached that of the bulk water.

**Figure 7:**
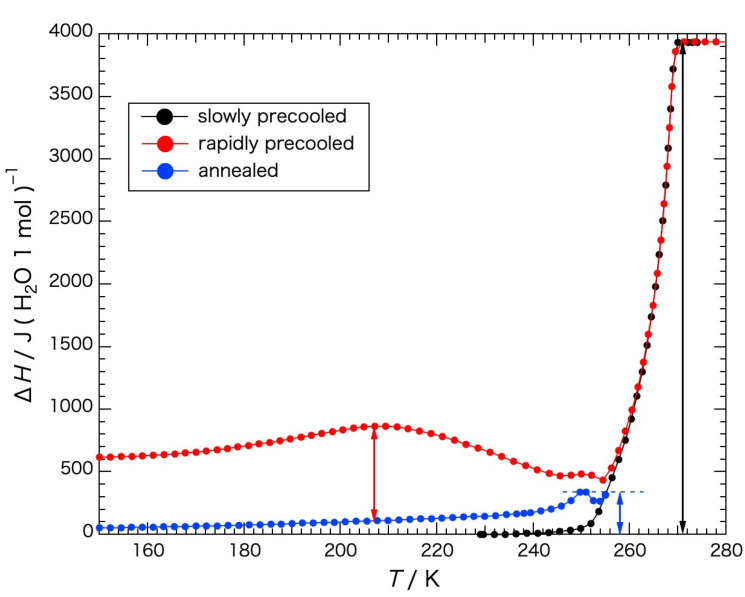
Fusion enthalpy of the water within the slowly precooled gel (●) and the crystallization enthalpy of the water during rewarming observed with the rapidly precooled gel (●) and the annealed gel (●). A red two−directional arrow indicates the crystallization enthalpy in the low subzero-temperature region, and a blue two-directional arrow indicates the crystallization enthalpy in the high subzero-temperature region.

For the determination of the fusion enthalpy (Δ*H*_fus_: the latent heat of the first-order phase transition) of the water within the gel, a baseline was drawn to the heat-capacity curve obtained with the slowly precooled gel (see the red dashed line (2) in Figure 5). Below 230 K the heat capacity of the slowly precooled gel was used as a baseline. From 230 K to the melting temperature the data of the heat capacity from 180 K to 230 K were extrapolated to the melting temperature by approximating the data with a straight line, and above the melting temperature the data from 272 K to 290 K were extrapolated to the temperature range below the melting temperature by approximating the data with a straight line. The melting temperature was regarded as the temperature where the slope of the temperature dependency of the fusion enthalpy was steepest, 269 K. Corresponding to the temperature below and above the melting temperature the two baselines were switched. The fusion enthalpies dependent on the temperature were obtained in the same way as the determination of the configurational enthalpy and are shown in Figure 7. In the case of the water in the gel precooled slowly, the fusion enthalpy, Δ*H*_fus_ = 3.93 kJ mol^−1^, corresponded to 82% of the configurational enthalpy (4.80 kJ (mol H_2_O)^−1^), which means that 82% of the configurational enthalpy was acquired by the fusion.

The enthalpy of ice crystallization during rewarming observed with the rapidly precooled and annealed gels corresponded to the excess configurational enthalpy on the basis of that observed with the gel precooled slowly. For the determination of the excess enthalpy of the water in both gels, it was necessary to obtain the heat-capacity data and to integrate the heat-capacity values from the baseline (the red dashed line (2) in Figure 5) dependent on the temperature. Then, the excess enthalpy in the temperature range to the initiation temperature of crystallization was obtained by the integration. For the temperature range above the initiation of crystallization, where the heat-capacity values were not determined because a large exothermic effect due to crystallization occurred, the excess enthalpy leading to the calculation of the crystallization enthalpy was derived as follows.

Assuming the spontaneous temperature-drift rate (d*T*/d*t*) was constant during the single cycle of the stepwise heating from the beginning of heating to the end of the temperature measurement (Δ*t*), Δ*T* can be expressed by the equation Δ*T* = (d*T*/d*t*) Δ*t*. Then, exothermic heat (Δ*H*_exo_) can be written as −Δ*H*_exo_ = *C_p_*_gross_ Δ*T*, where *C_p_*_gross_ is the total heat capacity, including that of the sample cell. Then, the total enthalpy change, Δ*H*_all_, can be written as Δ*H*_all_ = *E* − Δ*H*_exo_, where *E* is an applied electrical energy. Subtracting the contributions of the sample cell and Sephadex from the Δ*H*_all_, changing to the value per mole of water and reducing the contribution of the baseline value, the excess enthalpy was derived by the integration at each temperature of the measurement. The results are also shown in Figure 7. Note that the calculations were carried out assuming the excess enthalpies above the melting temperature were the same for the gels irrespective of the cooling mode, as all the water in the gel was in the liquid state.

In the case of the annealed gel, glass transition/crystallization and melting were observed in the high subzero-temperature region. However, they overlapped each other. In the case of the gel precooled rapidly, two glass transitions, i.e., a glass transition/crystallization in the low subzero-temperature region and a glass transition/crystallization and melting in the high subzero-temperature region, were observed, although the glass transition/crystallization and the melting in the high subzero-temperature region overlapped and were difficult to differentiate from each other. With knowledge of the overlap, the enthalpy of the ice crystallization was tentatively obtained. The enthalpy of the crystallization in the low subzero-temperature region corresponded to the difference between the peak value of the fusion enthalpy of the water in the rapidly precooled gel at 207 K and the value of the water in the annealed gel at the same temperature, 0.76 kJ mol^−1^, which is shown by the red two-directional arrow in Figure 7. The enthalpy of crystallization in the high subzero-temperature region was obtained using the annealed gel. The difference between the apparent peak value of the fusion enthalpy of the water in the annealed gel and the baseline was 0.34 kJ mol^−1^. These values correspond to 19% and 8.7% of the fusion enthalpy (3.90 kJ mol^−1^), respectively.

Water in the Sephadex^®^ G25 gel was classified in terms of the ice-crystallization behavior, as shown in Table 1. In the analysis, the value for the fusion enthalpy of ice, 6.01 kJ mol^−1^, and the values in Figure 5 and Figure 6 were used. The ratio of the non-crystallizing water in the gel was assumed to be the same for the slowly precooled and rapidly precooled gels. Then, the non-crystallizing water corresponding to 18% (100−82%) of the total water within the gel could be regarded as the hydration water. Crystallizing water during heating (rewarming) corresponded to 23% of the total water within the gel.

**Figure 8:**
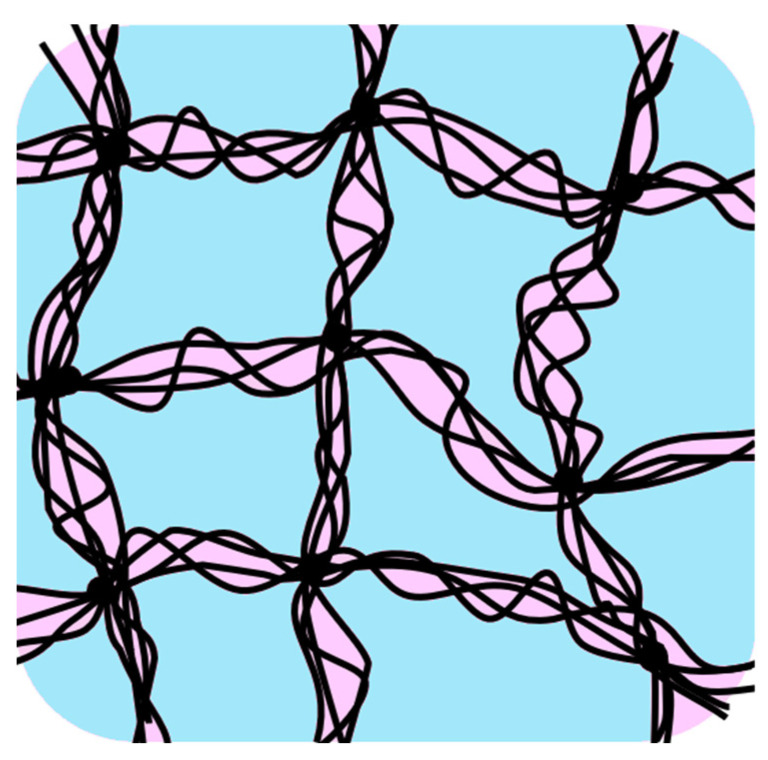
Schematic diagram of the network structure of Sephadex^®^ G25 gel (*h* = 1.00). Two types of molecular assemblies of water separated from each other are shown. Blue: compartmentalized by the network structure made of crosslinked dextran, pink: confined within the narrow spaces formed by the entangled dextran chains.

In Figure 8, a schematic diagram of the network structure of Sephadex^®^ G25 containing water of mass ratio *h* = 1.00 is shown. It is certain that the two glass transitions and ice crystallizations during rewarming observed in this study were caused by the different sites of the gel beads, as the glass-transition temperatures were very different from each other. Then, it can be considered that there were two types of molecular assemblies of water separated from each other. One was the water compartmentalized by the network structure made of crosslinked dextran (shown in blue) and the other was the water confined within the narrow spaces formed by the entangled dextran chains (shown in pink). These structures are shown in Figure 8.

Ice crystallization during rewarming observed in the high subzero-temperature region was due to the water trapped by the polymer network at the time of freeze initiation during precooling and remained unfrozen in a glassy state. The higher-temperature glass transition, observed at 240–242 K, was confirmed in the present study by the thermal-relaxation behavior shown in Figure 3C,D and the subsequent ice-crystallization exotherm. However, the temperature range of the glass transition was not as clearly overlapped with the endotherm due to the melting of small ice crystals and the exotherm due to the occurrence of ice crystallization triggered by the melting. The melting temperatures of small ice crystals are different depending on the crystal size, i.e., a smaller ice crystal will have a lower melting temperature. The complicated thermal behavior indicates that the ice crystals formed in the gel had a broad size distribution and that the water in the gel was vitrified when ice crystals of even the smallest size could not be formed.

Although both of the exotherms in the low subzero-temperature region (200–244 K) and in the higher-temperature region (250–255 K) indicating ice crystallization during rewarming were confirmed with the gel precooled rapidly, at 5 K min^−1^, it disappeared when precooled very slowly, at 20 mK min^−1^. This result suggests that there was a critical cooling rate for the appearance of the exotherm, which was determined by the competition between the rate of change in the polymer network at the time of freeze initiation during precooling and the rate of the diffusion of water molecules depending on the temperature.

The lower-temperature glass transition, observed at ca. 175 K, was probably due to the confined water within the narrow spaces formed by the entangled dextran chains, which was separated from the compartmentalized water and also from the confined water within the adjacent narrow spaces by the wall made of densely entangled dextran chains. Then, when crystallization was proceeding slowly at low temperatures during precooling, some of the confined water was trapped by the entangled dextran and turned into a glassy state, which crystallized during rewarming following the glass transition. As the water assemblies confined within the narrow spaces were small and separated from each other by the rigid wall made of the entangled dextran, they may have crystallized independently during rewarming in the broad temperature range at low subzero temperatures.

The anomalous ice-crystallization behavior during rewarming in polymer gels depends on the water content and the cooling rate as well as the compartment size, flexibility of the polymer chain, and continuity of the water phase between adjacent compartments that are inter-related via the density of crosslinks [3]. The effect of the cooling rate was made clear in this study. The water content is the most important factor for the appearance of the ice crystallization during rewarming, as it affects the swelling of the polymer-network structure. The state of the water in the gel controlled by the preparation method and the storage time after the sample preparation is also important in order to obtain reproducible results with such a heterogeneous sample as polymer gels.

## 3. Conclusions

Low-temperature thermal behavior of a Sephadex^®^ G25 gel was investigated using adiabatic calorimetry.

Although the exotherm in the high subzero-temperature region indicating the ice crystallization during rewarming was confirmed with the gel precooled rapidly at 5 K min^−1^, it disappeared when precooled slowly at 20 mK min^−1^. This result suggests that there is a critical cooling rate for the appearance of the exotherm in the high subzero-temperature range, which is determined by the competition between the rate of change in the polymer network at the time of freeze initiation during cooling and the rate of diffusion of water molecules at that time depending on the temperature.

Two glass transitions were observed during rewarming at ca. 175 K and 240–242 K. The higher-temperature glass transition due to the water trapped by the dextran network was confirmed by the thermal-relaxation behavior together with the appearance of a successive exotherm due to ice crystallization. Although it was not so clearly overlapped with an endotherm due to the melting of small ice crystals or an exotherm due to the occurrence of ice crystallization triggered by the melting, the result indicates that the ice crystals formed in the gel had a broad size distribution and that water in the gel was vitrified when ice crystals of even the smallest size could not be formed.

The lower-temperature glass transition was presumably due to the confined water within the narrow spaces formed by the entangled dextran chains. As the water assemblies within the narrow spaces were small and separated from each other, they may have crystallized independently during rewarming following the glass transition in the broad temperature range at low subzero temperatures.

The non-crystallizing water corresponding to the hydration water accounted for 18% of the total water within the Sephadex^®^ G25 gel. Crystallizing water during rewarming was found to correspond to 23% of the total water within the gel.

## 4. Materials and Methods

### 4.1. Materials

Sephadex^®^ G25 was obtained from GE Healthcare UK Ltd. G25 was dried under vacuum to obtain an anhydride. A calculated amount of distilled water was deposited on the anhydride under vacuum. The prepared samples were placed in a sealed container for at least one month to ensure uniformity and stability. The water content *h* (=g H_2_O/g dry G25) was determined from the mass change by drying the sample after the thermal measurement.

### 4.2. Differential Thermal Analysis (DTA)

The DTA experiments were performed from 100 to 300 K with a handmade apparatus [19,20]. It was essentially a metal block made of copper. Two wells were drilled symmetrically in the block, one being for the sample and the other for the reference material (α-alumina). The G25 sample was sealed with helium gas in a double-glazed sample tube and was inserted into the well. The block was covered with a glass vacuum jacket and measurements were carried out by introducing a small amount of helium gas. The temperature difference between the sample and the reference was measured by a chromel-constantan thermocouple inserted into the inner sample tube of the capillary structure. The mass of the sample used was 0.1–0.2 g. The heating rate was 2 K min^−1^ and the precooling rate was 5 K min^−1^.

### 4.3. Adiabatic Calorimetry

**Figure 9:**
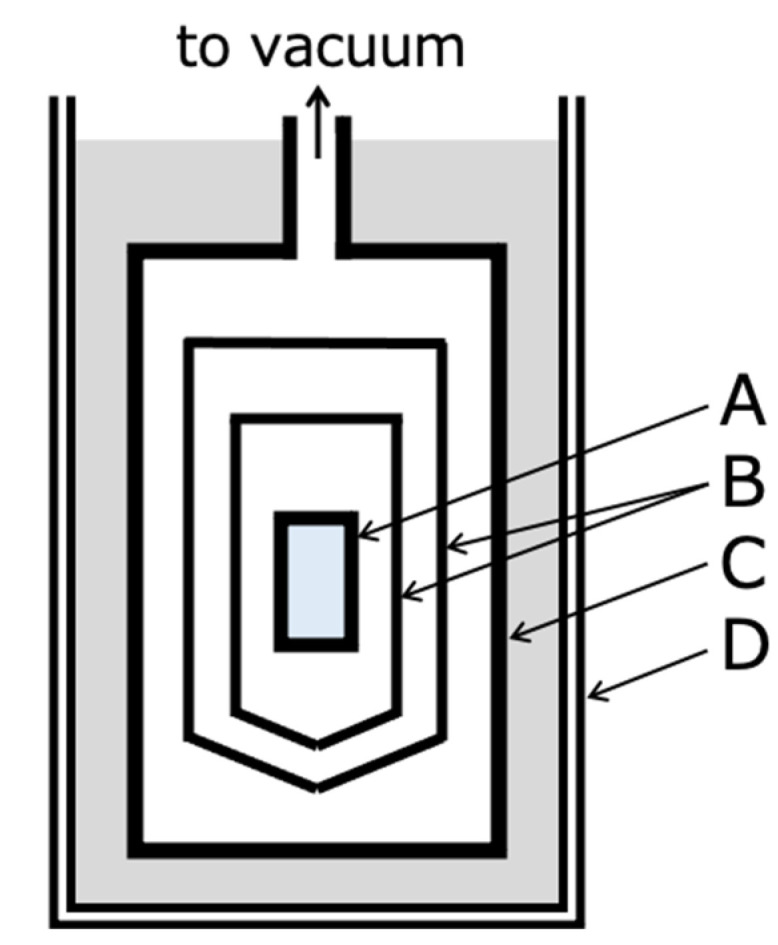
Schematic diagram of the adiabatic calorimeter cryostat used in this study. A: sample cell, B: double adiabatic shields, C: vacuum jacket, D: Dewar vessel.

The calorimetric measurements were carried out with an adiabatic calorimeter [21]. The calorimeter was developed for the simultaneous measurement of enthalpy and volume under high pressure, but measurements were carried out under ambient pressure using a sample cell for ambient pressure [22]. Figure 9 shows a schematic diagram of the adiabatic calorimeter cryostat used in this study. The sample is sealed in a sample cell under the He gas atmosphere. The sample cell (A) is under high vacuum in a vacuum jacket (C). Double adiabatic shields (B) around the sample cell are controlled at the same temperature as the sample to prevent heat leakage from the sample. When cooling the sample, it can be cooled down to 80 K by introducing a small amount of the He gas into the vacuum jacket with liquid nitrogen as a refrigerant in the Dewar vessel (D). The amount of sample loaded in the cell was 5.7373 g.

**Figure 10:**
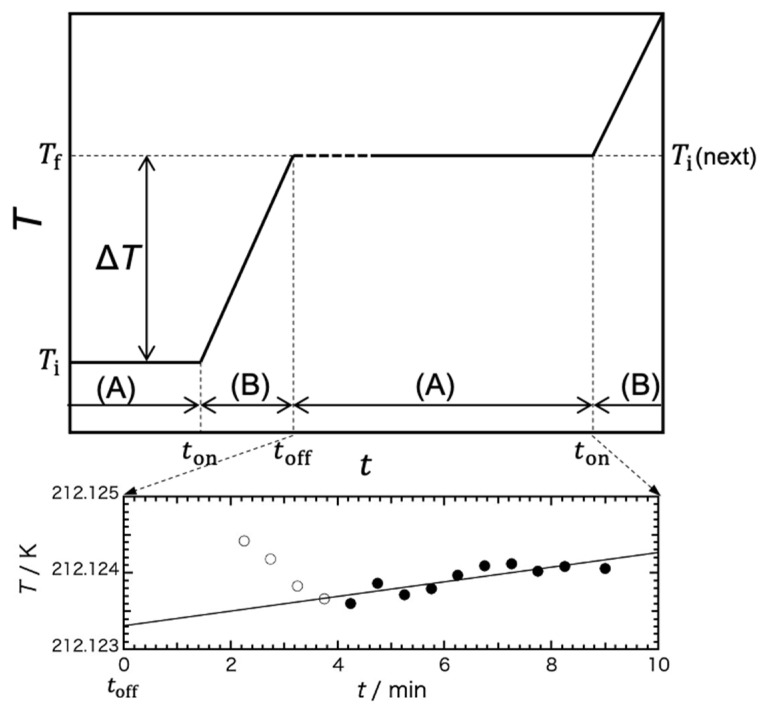
Schematic diagram of the temperature change of the sample with time during the heat-capacity measurement by using an adiabatic calorimeter (upper) and an example of the data of the temperature measurement (lower). The measurements were performed by the intermittent-heating method, repetition of an equilibration period (A) and a heating period (B). In the equilibration period (A), the dashed and solid lines represent thermal equilibration time and temperature measuring time, respectively.

Figure 10 shows a schematic diagram of the temperature change of the sample with time during the heat-capacity measurement with an adiabatic calorimeter. The heat-capacity measurements with an adiabatic calorimeter are performed by the intermittent-heating method, i.e., repetition of an equilibration period (A) and a heating period (B).

Under the adiabatic conditions, the temperature of the sample, which is in thermal equilibrium, is constant at *T*_i_. Here, the sample is heated by applying Joule heat Δ*E* from time *t*_on_ to *t*_off_ (heating period, B). After the heater is turned off, the sample reaches thermal equilibrium after an equilibration time (dashed line) of about 2–5 min, which is the time required for the temperature to become uniform and constant at temperature *T*_f_ (equilibration period, A). After the thermal-relaxation time, precise temperature measurements are performed for 5–10 min to determine the accurate *T*_f_, which is *T*_i_ in the next measurement. The gross heat capacity of the cell containing the sample is evaluated as *C*_gross_ = Δ*E/*Δ*T*, where the temperature rise, Δ*T = T*_f_ − *T*_i_, is typically 2–2.5 K.

The figure below shows an example of the measurement data. The temperature measurement starts 2 min after the heater is switched off and the sample is in a steady state (equilibrium state) from about 4 min. The temperature of the cell is measured with an Rh–Fe resistance thermometer (27 Ω at 273 K; Oxford Instrument Ltd., Abingdon-on-Thames, UK) calibrated on the temperature scale IPTS68 (30 < T < 300 K). Even in the steady state, the temperature rises slightly due to slight heat leakage between the sample cell and the adiabatic shield. The temperature-change rate d*T*/d*t*, obtained by least squaring the measurement points marked with black circles, was 5.8 mK h^−1^. This rate of temperature change was extrapolated to the time at the midpoint of the heating period (B) to obtain *T*_f_ (=212.1223 K) for this measurement and *T*_i_ (next) (= 212.1252 K) for the next measurement.

The heat capacity of the sample is evaluated by subtracting the heat capacity of the empty cell from the gross heat capacity of the cell. When a sample exhibits spontaneous exothermic or endothermic phenomena associated with melting, crystallization, or glass transition, they are observed as a rise or fall in the sample temperature within the equilibration period. Using the rate of temperature change of the rise or fall (d*T*/d*t*), the relaxation rate of the enthalpy of the sample is then evaluated as −(d*H*/d*t*) = *C*_gross_(d*T*/d*t*).

The glass transition is a phenomenon in which molecules freeze from a supercooled state to an unaltered configuration and move to a non-equilibrium state during the cooling process and return from a non-equilibrium state to an equilibrium state during the heating process. In adiabatic calorimetry, the latter enthalpy-relaxation phenomenon is observed. The relaxation time *τ*, which is the time required for the molecules to change configuration, increases with decreasing temperature. The glass transition is defined when the viscosity of a liquid is ~10^12^ Pa·s and the relaxation time is 10^2^–10^3^ s depending on the experimental time scale. Then, the temperature at which *τ* = 10^2^–10^3^ s is defined as the glass-transition temperature *T*_g_ [23,24,25,26].

Figure 4 shows schematically the typical temperature dependence of the configurational enthalpy (*H*_conf_) of the molecule near the glass-transition temperature (A) and the enthalpy-relaxation rate—(d*H*_conf/_d*t*) observed in the heat-capacity measurement (B). The configurational enthalpy *H*_conf_ is the enthalpy due to the spatial rearrangement of molecules. Compared to crystals, where the configuration does not change and only vibrational motion exists, the enthalpy of liquids is larger due to the contribution of molecular rearrangements. The dashed line in (A) indicates the equilibrium enthalpy.

In Figure 4A, a sample is heated after (a) rapid cooling from a temperature above *T*_g_ to a temperature below *T*_g_ at about 10 K min^−1^ and (b) slow cooling at about 10 mK min^−1^. The enthalpy of the quenched sample begins to deviate from the equilibrium enthalpy at a higher temperature and becomes constant at a high temperature compared to the slowly cooled sample. The heat capacity of the sample is measured from the lower temperature by the intermittent-heating method described above. The average heating rate in this case is about 0.1 K min^−1^.

In heating after rapid cooling (a), enthalpy relaxation is not detected at sufficiently low temperatures because the relaxation time is much longer than the measurement time, but then the enthalpy decreases to approach the equilibrium enthalpy and an exothermic effect is observed. When the equilibrium enthalpy line is reached, the relaxation time is still longer than the measurement time, so the enthalpy crosses the equilibrium line. Thereafter, an endothermic effect is observed as the temperature increases and as the enthalpy approaches the equilibrium line. At higher temperatures, the relaxation time becomes sufficiently short to coincide with the equilibrium line and the endothermic effect disappears. The temperature dependence of the enthalpy-relaxation rate in this process is shown in (a) of (B). The enthalpy-relaxation rate changes from positive to negative near *T*_g_.

In heating after slow cooling (b), the relaxation time is long enough at low temperatures that no appreciable relaxation effects can be observed until the enthalpy crosses the equilibrium line, and an endothermic effect is observed after the crossing. At higher temperatures, the enthalpy coincides with the equilibrium line and the endothermic effect disappears. The enthalpy-relaxation rate of this process is shown in (b) of (B). The enthalpy-relaxation rate of this endothermic effect shows a maximum near *T*_g_.

The existence of a glass transition can be established by observing these characteristic enthalpy relaxations. The Sephadex/water system is not a simple system that can be represented by a single relaxation time, and the environment of water molecules is diverse. Therefore, the temperature dependence as modeled in Figure 4 is not always observed.

Heat capacities and enthalpy-relaxation rates of the Sephadex^®^ G25 gel (*h* = 1.00) were measured from 80 to 300 K after precooling. During rapid cooling, He gas was introduced into the vacuum jacket (C in Figure 9) and the sample cell was cooled as quickly as possible. The cooling rate was therefore not controlled. Cooling rates were 5–6 K min^−1^ around 300–180 K, 4 K min^−1^ around 160 K, and 3 K min^−1^ around 140 K, so almost 5 K min^−1^. During slow cooling, the cooling was controlled adiabatically with the temperature of the adiabatic shields (B in Figure 9) set slightly lower than the sample cell and the cooling rate controlled at 20 mK min^−1^.

With the rapidly precooled samples, significant exothermic effects due to crystallization were observed in the two temperature ranges of 200–242 K and 250–255 K. However, as the two temperature ranges overlapped closely, the enthalpy changes of the two crystallizations could not be determined independently. Therefore, an annealing method was used to complete the crystallization at lower temperatures in order to observe only the crystallization at higher temperatures. Annealing was carried out with the rapidly precooled samples for 13 h at 237 K, which was within the temperature range of 200–244 K where the exothermic effect at the lower temperatures was observed with the rapidly precooled sample. At 237 K the crystallization rate was fast and melting did not occur even if the temperature was increased by 2–3 K during annealing. After annealing, the sample was cooled at 2–3 K min^−1^ and measurements started at 80 K. The average heating rate during heat capacity measurements by the intermittent heating method was 50–100 mK min^−1^.

## Figures and Tables

**Table 1:** Water in the Sephadex^®^ G25 gel (*h* = 1.00) classified in terms of the ice-crystallizing behavior during rewarming (%).

	Rapid Cooling (5 K min^−1^)	Slow Cooling (20 mK min^−1^)
Crystallizing during cooling	59%	82%
Crystallizing during heating *	23%	0%
Non-crystallizing	18%	18%

* Crystallizing during heating: 23% = 82 × (19 + 8.7)/100%; crystallizing during cooling: 59% = 100% − (23 + 18) %.

## Data Availability

Not applicable.
